# One-carbon metabolism factors and endometrial cancer risk

**DOI:** 10.1038/bjc.2012.534

**Published:** 2013-01-08

**Authors:** J J Liu, A Hazra, E Giovannucci, S E Hankinson, B Rosner, I De Vivo

**Affiliations:** 1 Department of Epidemiology, Harvard School of Public Health, Boston, MA 02115, USA; 2 Channing Division of Network Medicine, Department of Medicine, Brigham and Women’s Hospital and Harvard Medical School, Boston, MA 02115, USA; 3 Department of Medicine, Harvard Medical School, Boston, MA 02115, USA; 4 Department of Nutrition, Harvard School of Public Health, Boston, MA 02115, USA; 5 Department of Biostatistics, Harvard School of Public Health, Boston, MA 02115, USA

**Keywords:** endometrial cancer, epidemiology, one-carbon metabolism

## Abstract

**Background:**

This is the largest prospective cohort analysis to assess how dietary factors involved in one-carbon metabolism are associated with endometrial cancer incidence, using 26 years of follow-up data from the Nurses’ Health Study.

**Methods:**

The prospective cohort analysis of one-carbon metabolism dietary factors used the Cox proportional hazards model, and incorporated 788 incident endometrial cancer events from 1980 to 2006. Genotyping and unconditional logistic regression were performed on 572 endometrial cancer cases and their matched controls to examine 29 mostly non-synonymous single-nucleotide polymorphisms involved in one-carbon metabolism.

**Results:**

There were no significant dose–response relationships between intake of any of the one-carbon metabolism dietary factors and endometrial cancer incidence, but alcohol consumption of <1 drink a day was significantly protective (hazard ratio: 0.80; 95% CI: 0.68, 0.94). Those with the *MTHFR* 677 TT or *MTHFR* 1298 CC genotype had more protective associations for many of the dietary factors and endometrial cancer, but statistical power was limited in this analysis.

**Conclusion:**

Dietary levels of folate, choline, methionine, vitamin B2, vitamin B6 or vitamin B12 do not appear to influence endometrial cancer incidence. Moderate alcohol intake may protect against developing endometrial cancer.

Nutrients and genetic polymorphisms in the one-carbon metabolism pathway are involved in DNA methylation and synthesis, and therefore may have an important role in carcinogenesis. There is increasing evidence that abnormal DNA methylation contributes to endometrial carcinogenesis (Muraki *et al*, 2009; Tao and Freudenheim, 2010), suggesting that one-carbon metabolites and enzymes may influence the development of endometrial cancer. However, previous epidemiological studies on the associations between one-carbon metabolism dietary factors and endometrial cancer have yielded inconsistent findings (Negri *et al*, 1996; Jain *et al*, 2000; Xu *et al*, 2007; Kabat *et al*, 2008; Uccella *et al*, 2011). In addition, these studies did not examine the influence of choline and many potentially important single-nucleotide polymorphisms (SNPs), and several of them either focus only on a few nutrients or are not prospective. Therefore, we comprehensively assess the associations between dietary and genetic factors involved in one-carbon metabolism and endometrial cancer incidence in the Nurses’ Health Study (NHS), a large cohort providing 26 years of follow-up data.

## Materials and Methods

### Study population

The NHS is a prospective cohort study that began in 1976, when 121 700 female registered nurses aged 30–55 and residing in 11 US states completed an initial questionnaire. From 1989 to 1990, blood was collected from 32 826 participants.

For the cohort analysis, nurses who had hysterectomy, surgical menopause or cancers other than non-melanoma skin cancer were excluded at baseline (year 1980) and each subsequent follow-up cycle. There were 1 448 170 person-years of data involving 788 cases in the cohort analysis, covering 1980 to 2006.

For the nested case–control analysis, genotyping was performed on 572 cases and 572 matched controls from the NHS cohort. Matching factors included age and menopausal status. Controls were randomly selected up to and including the questionnaire cycle in which the case was diagnosed.

Completion of the self-administered questionnaire and submission of a blood sample were considered to imply informed consent. The NHS protocol was approved by the Human Research Committee of the Brigham and Women’s Hospital, Boston, MA, USA.

### Dietary exposure assessment

Intake level of one-carbon metabolism nutrients was calculated using validated food frequency questionnaire (FFQ) information as well as data from the US Department of Agriculture (U.S Department of Agriculture, 1989, 2004) and other sources (Zeisel *et al*, 2003). The cumulative average intake level involving information from 1980, 1984, 1986, 1990, 1994, 1998, 2002 and 2006 was used, adjusted for total energy intake.

### Endometrial cancer case ascertainment

Cases of invasive type 1 endometrioid adenocarcinoma, diagnosed between 1980 and 2006, were confirmed by medical record review. There were 788 eligible incident endometrial cancer cases during this time period who did not have missing dietary data. Among those cases, 572 had blood or buccal cell samples from which DNA was extracted for genotyping.

### Covariate information

Information on potential confounders was obtained from questionnaire responses that were updated every 2 years. Updated body mass index was calculated using height reported at baseline and weight reported at each cycle. Those missing weight in one cycle had their weight carried forward from the previous cycle, whereas those missing weight for two consecutive cycles were excluded until they again reported their weight. Smoking was quantified using pack-years.

### SNP selection and genotyping

The SNPs were selected because they are non-synonymous ones that are likely to affect protein functionality in one-carbon metabolism (Carr *et al*, 2009), or have a role in choline metabolism (Zeisel, 2008). The minor allele frequencies range from 0.04 to 0.47. Genomic DNA was extracted using protocol described previously (Prescott *et al*, 2010). The amount of missing genotyping data was <4%.

### Statistical analyses

The Cox proportional hazards model was used in the cohort analysis. The hazard ratio (HR) and 95% confidence interval (CI) were reported for risk of endometrial cancer in the categorical analysis. Tests for linear trend involved ordering the quintiles of the dietary factors and treating the values as continuous. The Anderson–Gill data structure was used to efficiently handle time-varying covariates (Therneau, 1997).

The unconditional logistic regression model was used in the nested case–control analysis involving SNPs in a Caucasian-only study sample, and the odds ratio was reported. The additive genetic model was used, which assumes that the effect of the heterozygous genotype is intermediate between the two homozygous genotypes. The homozygous genotype of the reference allele was coded as 0.

Analyses were done using the SAS Version 9.1 software (SAS Institute, Cary, NC, USA). Quintiles were created using the rank procedure. Multiplicative interaction terms involving continuous variables were created to test for effect modification using the Wald test. All *P*-values were two-sided.

## Results

In the multivariate cohort analysis, there were no significant dose–response relationships between intake of the one-carbon metabolism dietary factors and endometrial cancer incidence (Table 1). Women with alcohol intake in the second, third or fourth quintile had a significantly lower risk of endometrial cancer compared with women with the lowest quintile of intake. However, no association was observed among women in the top category of alcohol intake. Alcohol consumption of <1 drink (14 g) a day was significantly protective (HR: 0.80; 95% CI: 0.68, 0.94).

The results of the case–control analysis involving one-carbon metabolism SNPs are shown in Tables 2 and 3. The previously well-studied *MTHFR* 677 and *MTHFR* 1298 SNPs did not significantly modify the associations between total intake of the one-carbon metabolism dietary factors and endometrial cancer incidence (Table 2). However, for the *MTHFR* 677 SNP, those with the TT genotype had more protective associations for folate, vitamin B2, vitamin B6, vitamin B12 and alcohol intake, with the association reaching statistical significance for vitamin B6. For those with the *MTHFR* 1298 CC genotype, the associations were largely more protective compared with those with the other two *MTHFR* 1298 genotypes. The interaction between the other one-carbon metabolism SNPs and folate intake were examined, and we found significant effect modification of the association between folate and endometrial cancer by rs2276724 (*P*-interaction=0.01) and rs2886059 (*P*-interaction=0.03). Of the SNPs in this study, only rs202676 was significantly associated with endometrial cancer (*P*=0.03; Table 3).

## Discussion

In this large prospective study involving dietary factors in one-carbon metabolism and endometrial cancer with 26 years of follow-up, there were no significant associations between folate, choline, methionine, vitamins B2, B6, B12 and endometrial cancer incidence. Certain quantities of alcohol intake appeared to be protective against endometrial cancer. Although the *MTHFR* 677 and *MTHFR* 1298 SNPs did not significantly modify the associations between these dietary factors and endometrial cancer, the associations were suggestively more protective among those with the *MTHFR* 677 TT and *MTHFR* 1298 CC genotypes.

Our findings involving other one-carbon metabolism dietary factors are largely consistent with the results of previous studies (Negri *et al*, 1996; Jain *et al*, 2000; Xu *et al*, 2007; Kabat *et al*, 2008; Uccella *et al*, 2011). No previous studies have examined the association between choline and endometrial cancer, and our data suggest no association.

We found that alcohol intake below one drink a day significantly protected against endometrial cancer incidence, but there was no significant association for intake above one drink a day. This finding is remarkably consistent with the results from a recent meta-analysis involving seven cohort studies (Friberg *et al*, 2010). Among those consuming <1 drink a day, the meta-analysis found up to 7% decrease in endometrial cancer risk, whereas we found that the risk decreased around 20–30%.

We also examined whether genetic factors modified the associations between one-carbon metabolism dietary factors and endometrial cancer incidence. We found that despite no significant effect modification, those with the *MTHFR* 677 TT and *MTHFR* 1298 CC genotypes had more protective associations for many of the dietary factors and endometrial cancer. Another study also found a more protective association in those with the C allele for the *MTHFR* 1298 SNP when folate intake was high (Xu *et al*, 2007). For the other one-carbon metabolism SNPs examined, we found that rs2276724 and rs2886059 significantly modified the association between folate and endometrial cancer. Interestingly, both of these SNPs are on the *ALDH1L1* gene. We also found that rs202676, which is on the *FOLH1* gene, was significantly associated with endometrial cancer incidence. However, these findings were not statistically significant when accounting for the false discovery rate (Benjamini *et al*, 2001).

This is the first study involving endometrial cancer to examine the association with choline and many non-synonymous SNPs that may affect protein functionality in the one-carbon metabolism pathway. In addition, we have regularly updated information on dietary factors and many confounders, which minimises information bias and allows finer control of confounding. However, there are limitations in this analysis. First, the intake amount of the dietary factors in this cohort may not reflect that of other populations. Second, despite the detailed and regularly updated FFQ information, examining plasma biomarker levels of the one-carbon metabolism nutrients would be of interest. Finally, statistical power is limited in analyses involving the one-carbon metabolism SNPs, particularly for the interaction analyses.

Future studies involving other populations are necessary to validate our findings, especially for the lesser studied SNPs (e.g., the non-*MTHFR* SNPs) and nutrients (e.g., choline). It will also be informative to conduct a similar study in a population with higher intake of choline as well as plasma measurements of the nutrients.

## Figures and Tables

**Table 1:** Multivariate cohort analysis of one-carbon metabolism dietary factors and endometrial cancer incidence

	**Q1**	**Q2**	**Q3**	**Q4**	**Q5**	***P*** **-trend**
Total folate (μg day^−1^)^a^	275.8	402.0	509.7	623.8	794.0	
Events	123	174	163	172	156	
HR (95% CI)^b^	Ref	1.17 (0.93, 1.48)	1.11 (0.87, 1.41)	1.19 (0.94, 1.51)	1.10 (0.87, 1.41)	0.51
Folate from food only (μg day^−1^)^a^	214.3	282.7	336.5	396.4	493.9	
Events	127	146	165	168	182	
HR (95% CI)^b^	Ref	0.94 (0.74, 1.19)	1.01 (0.80, 1.28)	1.03 (0.81, 1.30)	1.12 (0.88, 1.41)	0.21
Total vitamin B2 (mg day^−1^)^a^	1.6	2.3	3.0	4.5	13.5	
Events	139	163	188	151	147	
HR (95% CI)^b^	Ref	1.01 (0.80, 1.27)	1.16 (0.93, 1.45)	0.99 (0.78, 1.25)	0.97 (0.77, 1.23)	0.75
Vitamin B2 from food only (mg day^−1^)^a^	1.2	1.5	1.8	2.1	2.6	
Events	118	166	142	203	159	
HR (95% CI)^b^	Ref	1.20 (0.94, 1.52)	1.02 (0.79, 1.30)	1.37 (1.09, 1.72)	1.06 (0.83, 1.35)	0.38
Total vitamin B6 (mg day^−1^)^a^	1.7	2.5	3.5	5.6	27.0	
Events	135	162	187	144	160	
HR (95% CI)^b^	Ref	1.01 (0.80, 1.27)	1.18 (0.94, 1.48)	0.95 (0.75, 1.21)	1.03 (0.81, 1.29)	0.94
Vitamin B6 from food only (mg day^−1^)^a^	1.3	1.6	1.9	2.2	2.7	
Events	128	148	147	175	190	
HR (95% CI)^b^	Ref	0.97 (0.76, 1.23)	0.91 (0.71, 1.15)	1.07 (0.84, 1.35)	1.12 (0.89, 1.41)	0.18
Total vitamin B12 (μg day^−1^)^a^	5.3	8.7	12.0	16.9	44.1	
Events	162	138	162	161	165	
HR (95% CI)^b^	Ref	0.81 (0.64, 1.02)	0.92 (0.74, 1.15)	0.91 (0.73, 1.14)	0.91 (0.73, 1.13)	0.77
Vitamin B12 from food only (μg day^−1^)^a^	3.5	5.0	6.3	8.0	10.8	
Events	143	157	146	170	172	
HR (95% CI)^b^	Ref	1.01 (0.80, 1.26)	0.90 (0.71, 1.13)	1.00 (0.80, 1.26)	1.03 (0.82, 1.29)	0.78
Total choline (mg day^−1^)^a^	227.3	281.5	323.6	370.6	444.8	
Events	136	154	165	177	156	
HR (95% CI)^b^	Ref	1.09 (0.86, 1.37)	1.12 (0.89, 1.41)	1.17 (0.94, 1.47)	1.02 (0.81, 1.29)	0.66
Total methionine (g day^−1^)^a^	1.2	1.5	1.7	2.0	2.4	
Events	144	151	170	151	172	
HR (95% CI)^b^	Ref	0.96 (0.76, 1.21)	1.07 (0.85, 1.34)	0.91 (0.72, 1.15)	0.98 (0.78, 1.22)	0.71
Total alcohol (g day^−1^)^a^	0.0	0.4	1.9	5.7	16.2	
Events	239	123	145	134	147	
HR (95% CI)^b^	Ref	0.74 (0.59, 0.92)	0.79 (0.64, 0.98)	0.80 (0.64, 0.99)	0.94 (0.76, 1.18)	0.49

Abbreviations: BMI=body mass index; CI=confidence interval; HR=hazard ratio.

a Median level of each quintile. Dietary reference intake levels for women 50–70 years old: folate: 400 μg day^−1^, vitamin B2: 1.1 mg day^−1^, vitamin B6: 1.5 mg day^−1^, vitamin B12: 2.4 μg day^−1^, choline: 425 mg day^−1^.

b Adjusted for calendar year (continuous), age (continuous, months), smoking (0, 0.1–20, 20.1–40, >40 pack-years), BMI (continuous, kg m^−2^), race (White, Black, others), age at menarche (7–11, 12, 13, 14–18 years), oral contraceptive use (no use, <1, 1–3, 3–6, >6 years), menopausal status (premenopausal, postmenopausal), postmenopausal hormone use (no use, oral conjugated oestrogen, oral oestrogen and progesterone, others), and parity (0, 1, 2, 3, >3).

**Table 2:** Multivariate analysis of one-carbon metabolism dietary factors and endometrial cancer incidence by *MTHFR* genotypes^a^

	***MTHFR*** **677 (OR (95% CI))**^b^			
	**CC (** ***N*** **=484)**	**CT (** ***N*** **=506)**	**TT (** ***N*** **=129)**	***P*** **-interaction**
Folate	0.79 (0.52, 1.20)	1.32 (0.89, 1.95)	0.63 (0.25, 1.60)	0.88
Choline	1.52 (1.02, 2.27)	1.31 (0.88, 1.95)	1.37 (0.58, 3.27)	0.99
Methionine	1.02 (0.68, 1.53)	0.92 (0.62, 1.36)	1.20 (0.52, 2.78)	0.60
Vitamin B2	1.05 (0.70, 1.59)	0.98 (0.67, 1.44)	0.50 (0.19, 1.29)	0.40
Vitamin B6	1.02 (0.68, 1.55)	1.30 (0.88, 1.92)	0.30 (0.11, 0.83)	0.37
Vitamin B12	1.16 (0.77, 1.74)	1.02 (0.69, 1.52)	0.52 (0.19, 1.41)	0.28
Alcohol	1.48 (0.97, 2.28)	0.79 (0.53, 1.19)	0.65 (0.26, 1.64)	0.07

	***MTHFR*** **1298 (OR (95% CI))**^b^			
	**AA (** ***N*** **=498)**	**AC (** ***N*** **=506)**	**CC (** ***N*** **=121)**	***P*** **-interaction**
Folate	1.09 (0.73, 1.61)	0.89 (0.60, 1.33)	0.45 (0.13, 1.57)	0.19
Choline	1.44 (0.98, 2.13)	1.47 (0.99, 2.19)	0.80 (0.27, 2.39)	0.30
Methionine	0.96 (0.65, 1.42)	1.08 (0.73, 1.61)	0.66 (0.21, 2.06)	0.84
Vitamin B2	0.91 (0.61, 1.34)	1.03 (0.70, 1.53)	0.36 (0.11, 1.20)	0.43
Vitamin B6	0.88 (0.59, 1.30)	1.08 (0.72, 1.61)	0.44 (0.14, 1.42)	0.63
Vitamin B12	0.92 (0.61, 1.37)	0.92 (0.62, 1.36)	0.96 (0.30, 3.12)	0.68
Alcohol	0.82 (0.55, 1.23)	1.03 (0.67, 1.56)	1.77 (0.62, 5.07)	0.17

Abbreviations: BMI=body mass index; CI=confidence interval; OR=odds ratio.

a Dietary factors were dichotomised at the median. ORs indicate the association magnitude for above versus below median intake, stratified by genotype. The sample sizes for the genotypes include both cases and controls.

b Adjusted for age, smoking, BMI, race, age at menarche, oral contraceptive use, menopausal status, postmenopausal hormone use and parity.

**Table 3:** Associations between one-carbon metabolism SNPs and endometrial cancer incidence

				**Endometrial cancer** b	
**SNP**	**Gene**	**MAF**	**Reference allele** a	**OR (95% CI)**	***P*** **-value**
rs1127717 (C,T)	*ALDH1L1*	0.20	C	0.97 (0.79, 1.19)	0.78
rs4646750 (C,T)	*ALDH1L1*	0.07	C	1.07 (0.78, 1.47)	0.68
rs2886059 (A,C)	*ALDH1L1*	0.16	A	1.00 (0.79, 1.26)	0.99
rs2276724 (C,T)	*ALDH1L1*	0.15	C	0.91 (0.72, 1.14)	0.41
rs2372536 (C,G)	*ATIC*	0.32	C	1.04 (0.88, 1.24)	0.65
rs3733890 (A,G)	*BHMT*	0.30	A	1.00 (0.83, 1.19)	0.96
rs9001 (G,T)	*CHDH*	0.05	G	1.14 (0.78, 1.67)	0.49
rs12676 (A,C)	*CHDH*	0.30	A	0.87 (0.73, 1.03)	0.10
rs1021737 (G,T)	*CTH*	0.29	G	0.85 (0.71, 1.02)	0.08
rs2228612 (C,T)	*DNMT1*	0.06	C	0.95 (0.67, 1.35)	0.78
rs202676 (T,C)	*FOLH1*	0.22	T	1.26 (1.03, 1.54)	0.03
rs8788 (C,T)	*GART*	0.17	C	0.99 (0.79, 1.24)	0.93
rs8971 (C,T)	*GART*	0.24	C	0.90 (0.74, 1.09)	0.29
rs11545077 (C,T)	*GGH*	0.25	C	1.15 (0.96, 1.37)	0.13
rs11545078 (A,G)	*GGH*	0.10	A	0.87 (0.65, 1.15)	0.32
rs2236225 (A,G)	*MTHFD1*	0.45	A	1.02 (0.87, 1.21)	0.79
rs1950902 (A,G)	*MTHFD2*	0.18	A	0.97 (0.78, 1.19)	0.75
*MTHFR* 677 (C,T)	*MTHFR*	0.34	C	1.06 (0.89, 1.27)	0.50
*MTHFR* 1298 (A,C)	*MTHFR*	0.33	A	1.03 (0.87, 1.23)	0.71
rs1805087 (A,G)	*MTR*	0.19	A	1.03 (0.84, 1.26)	0.79
rs1801394 (A,G)	*MTRR*	0.47	A	1.10 (0.93, 1.30)	0.25
rs1532268 (C,T)	*MTRR*	0.37	C	0.98 (0.82, 1.16)	0.78
rs2287780 (C,T)	*MTRR*	0.04	C	0.73 (0.47, 1.14)	0.16
rs16879334 (C,G)	*MTRR*	0.04	C	0.79 (0.50, 1.24)	0.30
rs162036 (A,G)	*MTRR*	0.11	A	1.27 (0.97, 1.67)	0.09
rs10380 (C,T)	*MTRR*	0.09	C	1.19 (0.89, 1.59)	0.25
rs7946 (T,C)	*PEMT*	0.25	T	0.96 (0.80, 1.15)	0.68
rs1979277 (A,G)	*SHMT1*	0.29	A	0.87 (0.73, 1.03)	0.11
rs1051266 (C,T)	*SLC19A1*	0.44	C	0.97 (0.82, 1.14)	0.68

Abbreviations: CI=confidence interval; MAF=minor allele frequency; OR=odds ratio; SNP=single-nucleotide polymorphism.

a The homozygous genotype of the reference allele was coded as 0.

b Adjusted for the matching factors age and menopausal status.
